# Exosomes: Potential Therapies for Disease via Regulating TLRs

**DOI:** 10.1155/2020/2319616

**Published:** 2020-05-27

**Authors:** Hong-Yan Guo, An-Chun Cheng, Ming-Shu Wang, Zhong-Qiong Yin, Ren-Yong Jia

**Affiliations:** ^1^Research Center of Avian Disease, College of Veterinary Medicine of Sichuan Agricultural University, Wenjiang District, Chengdu 611130, China; ^2^Institute of Preventive Veterinary Medicine, Sichuan Agricultural University, Wenjiang District, Chengdu 611130, China; ^3^Key Laboratory of Animal Disease and Human Health of Sichuan Province, Wenjiang District, Chengdu 611130, China

## Abstract

Exosomes are small membrane vesicles that retain various substances such as proteins, nucleic acids, and small RNAs. Exosomes play crucial roles in many physiological and pathological processes, including innate immunity. Innate immunity is an important process that protects the organism through activating pattern recognition receptors (PRRs), which then can induce inflammatory factors to resist pathogen invasion. Toll-like receptor (TLR) is one member of PRRs and is important in pathogen clearance and nervous disease development. Although exosomes and TLRs are two independent materials, abundant evidences imply exosomes can regulate innate immunity through integrating with TLRs. Herein, we review the most recent data regarding exosome regulation of TLR pathways. Specifically, exosome-containing materials can regulate TLR pathways through the interaction with TLRs. This is a new strategy regulating immunity to resist pathogens and therapy diseases, which provide a potential method to cure diseases.

## 1. Introduction

The immune system is a tool of creatures that go against pathogen invasion and eliminate pathogens, which includes two components: innate immunity and acquired immunity. Innate immunity is the first barrier of host defense that resist pathogens while the acquired immunity is involved in eliminating pathogens. To date, both composes have been studied well. Acquired immunity is characterized by antigen-specific receptors, which are used to recognize nonself and the major process of nonself-recognition, such as diversity, clonality, and memory. However, this knowledge is limited to mammals, and how nonself-recognition process works in less evolved organisms is not entirely clear. Innate immune system is the first line to resist pathogens, and germline-encoded pattern recognition receptors (PRRs) serve a role to recognize microorganisms. PRRs are expressed on all cells of a given type and can recognize pathogen-associated molecular patterns (PAMPs), a microbial component that is essential for a microorganism survive and not easy to alter regardless of their life cycle stage. The mechanism of innate immunity is common. Firstly PRRs interact with specific PAMPs and then activate specific signaling pathways, lead to distinct antipathogen responses [1].

Toll-like receptors (TLRs) are an important member of PRRs, which can sense organisms like bacteria, virus, parasite, and fungi [2, 3] and then promote the synthesis and release of a variety of inflammatory cytokines and chemokines [4, 5]. Toll was first discovered in *Drosophila* in the 20th century [6]; then, twelve mice, ten humans [7], and ten avian functional TLRs [8] were identified. TLRs are type I transmembrane proteins [7] containing two domains, in which extracellular domains contain varying numbers of leucine-rich-repeat (LRR) motifs and a cytoplasmic signaling domain is termed the Toll/IL-1R homology (TIR) domain [9]. TLRs express on various immune cells like macrophages, dendritic cells (DCs), B cells, specific types of T cells, and even on nonimmune cells such as fibroblasts and epithelial cells [1]. Furthermore, TLRs expressed extra- or intracellularly and the distribution of TLRs is related to their function. To be specific, TLRs 1, 2, 4, 5, and 6 are expressed on the cell surface, while TLRs 3, 7, 8, and 9 are found in intracellular compartments [1]. TLR2 can recognize various microbial components including lipoproteins/lipopeptides, peptidoglycan, glycosylphosphatidylinositol, phenol-soluble modulin, zymosan, and glycolipids [10]. However, this wide spectrum of microbial components of TLR2 is functionally associated with TLR1 and TLR6 [11–13]. TLR3 can recognize double-stranded RNA (dsRNA) produced from many viruses [14], while TLR7 can recognize GU-rich single-stranded RNA (ssRNA) [15], syntheticpoly(U) RNA, and certain small interfering RNAs [16]. TLR8 has high homology to TLR7, but only human TLR8 can recognize single-stranded RNA (ssRNA) virus [17]. TLR5 recognizes bacterial flagellin [18] and TLR9 is a receptor for CpG DNA [19], but the specific function of TLR10 remains elusive [20].

**Figure 1:**
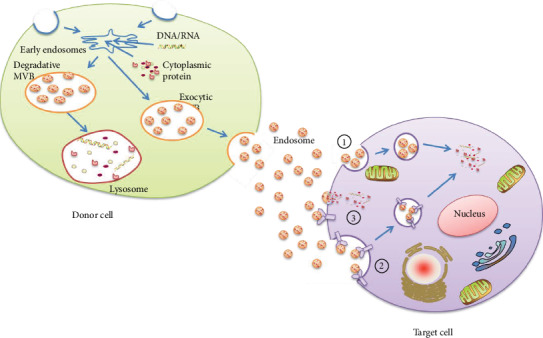
The formation and transportation of exosome. The formation of a mature exosome is closely related to two processes. First, the limiting membrane is invaginated to form multivescular bodies (MVB). Second, a part of MVBs called exocytic MVBs bud off into the lumen of the late endosome to form the second membrane and then secrete into the extracellular space along with their cargo, while another part of MVBs evolve into lysosomes for degradation called degradative MVBs. Then, exosomes are translated by body fluid and the three ways for receipt cell to take up exosomes are ➀ endocytosis, ➁ lipid raft-mediated internalization, and ➂ by combining with the target cell membrane directly.

Except pathogens, exosomes can also activate the TLR signaling pathway. Exosomes is a type of small membrane vesicles (30~150 nm in diameter) with lipid bilayers [21] belonging to extracellular vehicles (EVs) [22]. Exosomes originate from the endocytic route and are formed by the inward budding of the plasma membrane [23]. Firstly, limiting membrane invagination was translated into intraluminal vesicles (ILVs) (also called multivesicular bodies (MVB)) to form the first membrane, from when two types of MVB formed. The first one is exocytic MVB, which can bud off into the lumen of the late endosome, to form the second membrane [24, 25], and is secreted into the extracellular space along with their cargo, we called it exosomes [26]. And the second type of MVB is degradative MVB which evolve into lysosomes for degradation [25]. Because of the variety origin of exosomes and its special form process, the protein of exosomes depends on the parent cell [27, 28]; however, some basic structures like transmembrane proteins (Mac-1*α* chain, MHC-II*β* chain, and CD9), cytoplasmic protein (hsc73, annexin II, and Gi2*α*), and milk fat globule-EGF factor 8 protein (MFG-E8) are conserved [29]. Exosomes encapsulate multiple proteins and materials such as nucleic acid like transforming growth factor-*β* (TGF-*β*) [30], genomic DNAs [31], and microRNAs (miRNAs) [32], and exosomes almost exist in all body fluids [27, 28]; this makes them serve a primary role in intercellular communication. For now, three ways were observed for an objective cell to take up exosomes, which are endocytosis [33–35], lipid raft-mediated internalization [36], and by combining with the target cell membrane directly [37] (Figure 1).

## 2. Exosomes Regulate the Role of TLRs in Neurological Diseases

TLRs are a crucial immunity factor in neurological diseases which can induce abundant inflammatory cytokines and target the inflammatory response. The inflammatory reaction in nerves is special; it will perform neuroprotection when it consists of short time; otherwise, it will result in neurodegeneration [38]. According to the reports, TLRs are involved in various neurological diseases including Alzheimer's disease (AD), Parkinson's disease (PD), amyotrophy lateral sclerosis (ALS), and stroke.

Alzheimer's disease (AD) is a progressive neurodegenerative illness diagnosed clinically by the presence of extracellular neuritic plaques in the limbic brain regions and intracellular neurofibrillary tangles [39]. The primary component of neuritic plaques is amyloid-beta (A*β*). A*β* has two forms, a monomeric form and insoluble fibrillar form [40], in which fibrillar forms of A*β* are toxic to neurons [41]. According to the research, fibrillar forms of A*β* can trigger the TLR2, TLR4, and TLR9 signaling pathways to induce microglial inflammatory response [42, 43], which can then attenuate the symptoms of AD at the early stage [43]. Lately, researchers found that the brain exosomes has abundant A*β,* and continuing to infuse mice with these exosomes for two weeks could decrease the expression of A*β*, inhibit amyloid deposition, and attenuate synaptic toxicity [44, 45]. This suggests that A*β*-abundant brain exosome can activate the TLR2, TLR4, and TLR9 signaling pathways to attenuate the symptoms of AD at the early stage. However, when AD gets worse, inhibiting the expression of TLR2 via interacting with exosome miR-146a could attenuate microglial activation and amyloid accumulation [46]. These results suggest that injecting A*β*-abundant exosomes might be a potential method to cure AD at the early stage, while giving exosome miR-146a might be a potential way to attenuate the symptoms of AD at the late stage.

ALS is a chronic neurodegeneractive disease expressing muscle atrophy, paralysis, and death resulting from loss of motor neurons [47]. Even the mechanisms involved in this selective degeneration is not clear, recent research explored the interaction between motor neurons and glial cells in mouse models of familial ALS-expressing forms of mutant copper-zinc superoxide dismutase (SOD1) give us a clue [48]; it reveals that astrocytes play a major protective role during ALS. Letiembre et al. explored that SOD1-abundant exosome can upregulate the expression of TLR2 to trigger microglial neurotoxic inflammatory responses [49, 50], while Pinto et al. found that exosome miR-124 could increase the expression of TLR4 to trigger spinal cord astroglial and microglial reaction and enhance spinal motor neuron loss in the ALS [51, 52]. These results imply that repressing the expression of TLR2 and TLR4 is a potential way to relieve the symptoms of ASL, a effective way to eliminate SOD1-abundant exosome and exosome miR-123 would be beneficial in the treatment of AD.

Alpha-synuclein (*α*-syn) is a cellular hallmark of PD [53]; it can form toxic oligomeric intermediates directly linked to neuronal damage [54]. As reports say, the toxic effect of *α*-syn can hurt inducing cells or neighboring cells through exosomes [55, 56]. According to the results, the interaction between exosome *α*-syn and TLRs is beneficial to cure PD. For one thing, extracellular *α*-syn can increase the expression of TLR1, TLR2, TLR3, and TLR7 [57, 58] and then trigger an inflammatory reaction. For another thing, TLR4 was identified as a trigger of PD pathogenesis [59], which can promote cells inducing exosome *α*-syn. Thus, the absence of TLR4 reducing the number of *α*-syn in neurons results in a low level of neuroinflammation and neurotoxin associated with PD [60]. Although giving extracellular *α*-syn can triggerneuroinflammation, prolonged neuroinflammation can damage the neurons. Therefore, inhibition of TLR4 expression is an ideal way to reduce PD symptoms, while exosomes are an ideal way to present drugs or chemical compounds in the brain.

Stroke is a prevalence that augments with aging population and marked by adult disability [61]. According to the results, TLR4, TLR7, and TLR8 are all involved in stroke, in which TLR4 and TLR8 have detrimental roles in ischemic stroke [62, 63], while the activation of TLR7 can induce robust neuroprotection against stroke by triggering a novel type I interferon-mediated mechanism [64]. Unfortunately, there are no studies demonstrating the effects of exosome on TLRs, but the fact that exosome miR-21 was highly expressed in patients with stroke and exosomal miR-21 could activate TLR8 signaling directly a potential negative function of exosome miR-21 in stroke [65]. However, more studies are required.

## 3. Exosomes Regulate the Role of TLRs in Cancer

Tumor development and metastasis are closely related to tumor microenvironment, thus a continuous crosstalk between cancer cells and other cellular components is required to sustain tumor progression [66]. Thus, exosome, a novel way of cell communication, plays an important role in cancer development process.

Mesenchymal stem cells (MSCs) are an important component in tumor microenvironment, which is evidenced by tumor-supporting roles of tumor including lung tumor. The recent research illuminates that exosomes derived from A549 lung tumor cells could trigger a proinflammatory phenotype in MSCs via the TLR2 signaling pathway, then promoting lung tumor growth in a mouse xenograft model [67]. Contrary to TLR2, the activation of TLR7 by tumor-derived exosomes miR-21, miR-27b, and miR-29a can effectively inhibit the development of lung cancer [68]. What intrigued us is tumor -derived exosome has dual functions, promoting or inhibiting the growth of tumor. This makes us curious which function of exosomes plays a major role in the course of disease, or if in different stages of disease exosome play different roles? Only when we understand deeper the functional rules of exosomes can we better use the relationship between exosomes and TLRs for the treatment of cancer. Therefore, more experiments are required.

Except lung tumor, the interaction between exosomes and TLRs is also observed in ovarian cancer and pancreas cancer. In ovarian cancer, the cancer cell-derived exosomes could trigger inflammatory responses to go against cancer via the TLR2 and TLR4 signaling pathways [69]. While the exosomes derived from pancreas cancer cells can downregulate the expression of TLR4 via miR-203, inhibiting the role of TLR4 in promoting angiogenesis in pancreatic cancer [70]. Learning from these results, we can see that the interaction between the exosome and TLRs has a dual effect on the development of cancer, and understanding this knowledge will help us to effectively use the relationship between exosome and TLRs to treat diseases.

## 4. Exosomes Regulate the Role of TLRs in Other Diseases

Except neurological diseases and cancer, the immunimodulatory function of exosomes is also observed in other disease and physiological process. As the first line of host, TLRs play an important role against pathogen invasion including viruses. There are many ways for a virus to trigger the TLR signaling pathway; using exosome to transfer viral materials is one of them. For example, Rift Valley fever virus (RVFV) can integrate their viral RNA into exosomes and then trigger the TLR pathway through exosome transport [71]. Moreover, after Mycobacterium avium glycopeptidolipids- (M. avium-) infected macrophages, the exosomes derived from macrophages contain M. avium glycopeptidolipids (GPLs), which then can trigger proinflammation via TLR2 and TLR4 [72]. In addition, the virus can change the material expression in exosomes. For example, miR-148a-5p can target and downregulate the expression of TLR3. However, the expression of miR-148a-5p in exosomes is supressed following Duck Tembusu Virus (DTMUV), which in turn promotes TLR3 expression to resist DTMUV infection [73].

Taking exosomes as a tool to trigger TLR pathways has also been observed in other diseases. Transactivating response element RNA (TAR RNA) is a main material of exosomes that was induced from primary cell-infected HIV-1, which can bind to TLR3 effectively and release various of inflammatory factors to resist HIV-1 invasion [74]. Exosomes derived from lymphocytic leukemia (CLL) can target TLR7 signaling to promote innate immunity [75], while exosomes isolated from systemic lupus erythematosus (SLE) patients' serum can produce abundant IFN-*α*, TNF-*α*, IL-1*β*, and IL-6 via the TLR1/2, TLR7, TLR9, and TLR4 pathways [76].

What intrigues us is that the activation of the TLR pathway through exosomes can also be a marker of pregnancy in ruminants. According to Ruizgonzález et al.'s research, the exosomes isolated from uterine flushing during pregnancy can act with oTr1 to target the TLR-mediated signaling (especially TLR7 and TLT8 signaling) which then produce abundant IFNT to indicate pregnancy in ruminants [77]. Syphilis' research found that miR-216a-5p-containing exosomes significantly attenuated the rTp17-induced inflammatory response by targeting TLR4 [78]. Moreover, TLR2-induced megakaryocytes release extracellular vesicles (EVs) that are able to recapitulate TLR2 signaling in the megakaryocytic cell line (Dami cells) to replenish the immune effector [79].

## 5. Conclusions and Future Perspectives

Innate immunity is the first barrier of the host to resist pathogens, which can be regulated by various materials. Exosomes, as a tool of transmitting information between cells, play a role in regulating the TLR signaling pathway. In this review, we have reviewed the most recent data regarding exosome regulation of the TLR pathways, which provide a potential method to cure diseases. According to our review, we found that exosomes can not only trigger innate immunity but also inhibit innate immunity. At the same time, we found that triggering the innate immunity is not necessarily beneficial to the disease, or even makes the disease worse. Therefore, understanding the interaction between exosome and TLRs and their results is important for developing new therapeutic drugs, vaccines, and a novel way to detect diseases.

According to our review, the research focuses on exploring the interaction between exosomes and TLRs are limited because they only explained how exosome materials regulate the TLR pathway, but how exosomes surface materials influence the expression of TLRs remains to be explored. Known from previous research, the most exosome materials and surface proteins come from parent cells [27, 28]; thus, the composition of an exosome is complex and special, and this means different cell-derived exosomes have different functions on regulating TLRs. In fact, this phenomenon is common. For example, ovarian cancer cell-derived exosomes could trigger the TLR2 and TLR4 signaling pathways [69], but exosomes induced from primary cell-infected HIV-1 could activate TLR3 [74]. Moreover, we found even the same exosome will have more than one function. For example, A549 lung tumor cell-derived exosomes can trigger the TLR2 and TLR7 signaling pathway, but the action of TLR2 can promote lung tumor growth [67], while the activation of TLR7 can effectively inhibit the development of lung cancer [68]. And because of this feature of exosome, the regulation of TLRs by an exosome becomes complex. Therefore, in order to better utilize the relationship between exosomes and TLRs to treat diseases, it is important to deeply understand when and what function of exosomes will play during the disease. However, at present, the research on the regulation of TLRs by an exosome is still in the basic stage. Thus, to further explore the interaction between exosome and TLRs, to detect what materials a specific exosome contains and how these materials change during disease is a good start. In addition, the present studies of exosomes were limited to cultured cells; the functions of exosome in vivo remain to be elucidated. This is because the technology to explore the biological function of exosomes in vitro is not widely available, but some experiments have studied the functions of exosomes in living animals, but this is only limited to the parts of living animals and fundamentals [80]. Thus, to further explore the biological function of exosomes in vitro is required.

In addition to developing techniques for studying exosomes, understanding the roles of exosomes and finding a reliable way to give medicine or chemical compound are also important. Up to now, we have some methods of administration, such as intramuscular injection, oral administration, and eye drops. But with each method having its own disadvantages, specially some drugs cannot cross the blood-brain barrier. However, an exosome as a tool to transfer materials in vitro, it could be an ideal tool to transfer medicine and chemical components, especially since it can cross the blood-brain barrier. In fact, research has evidenced that using exosomes as a tool to deliver drugs or chemical components for treatment or attenuating disease symptoms. For example, studies have shown that injecting mice with an exosome carrying a specific siRNA can successfully reduce the level of *α*-syn [81]. Learning from our review, exosome materials could regulate the development of diseases through interacting with TLRs. Using this knowledge, integrating materials into these exosomes and injecting them into the body is a viable therapeutic approach, while an efficient and rapid technique for integrating active factors into exosomes and delivering them to specific sites is necessary too.
